# Prevention of Non-Contact Anterior Cruciate Ligament Injuries among Youth Female Athletes: An Umbrella Review

**DOI:** 10.3390/ijerph19084648

**Published:** 2022-04-12

**Authors:** Anmol T. Mattu, Brianna Ghali, Vanessa Linton, Alex Zheng, Ian Pike

**Affiliations:** 1 MD Undergraduate Program, Faculty of Medicine, University of British Columbia, Vancouver, BC V6T 1Z3, Canada; 2 Undergraduate Medical Education, Cumming School of Medicine, University of Calgary, Calgary, AB T2N 4N1, Canada; bmghali@ucalgary.ca; 3 BC Injury Research and Prevention Unit, BC Children’s Hospital Research Institute, Vancouver, BC V6H 3V4, Canada; vanessa.linton@bcchr.ca (V.L.); alex.zheng@bcchr.ca (A.Z.); ipike@bcchr.ca (I.P.); 4 Department of Pediatrics, Faculty of Medicine, University of British Columbia, Vancouver, BC V6H 3V4, Canada

**Keywords:** anterior cruciate ligament (ACL), female athlete, youth, injury prevention, prevention program, neuromuscular training, meta-analysis

## Abstract

Anterior cruciate ligament (ACL) injuries account for a large percentage of knee injuries, disproportionately affecting female athletes. To help health professionals stay current, we performed an umbrella review to evaluate the effectiveness of ACL injury prevention programs in reducing non-contact ACL injury rates, determine the effective components within interventions, and provide clinical recommendations. Twelve databases (Medline, Embase, Cochrane Database of Systematic Reviews, SPORTDiscus, Cumulative Index to Nursing and Allied Health Literature, PEDro, Web of Science Core Collection, Epistemonikos, TRIP, BC Guidelines and Protocols, CPG Infobase, ProQuest Dissertations and Theses Global) were searched in May 2021 to identify relevant systematic reviews and meta-analyses. Four databases were searched again in September 2021 to identify recent primary literature. Non-contact ACL injury data were extracted to calculate incidence rate ratios (IRRs) and these were combined using an inverse variance random-effects model. A qualitative assessment of included reviews was performed. The methodological quality of the studies was assessed using a Measurement Tool to Assess Systematic Reviews 2 (AMSTAR 2) or Cochrane Risk-of-Bias Tool for Randomized Trials (RoB 2). Sixteen reviews and two primary studies met the inclusion criteria. Across 11 primary studies, prevention programs were effective in reducing non-contact ACL injuries by 64% (IRR = 0.36 (95% CI: 0.18–0.70)). A multi-faceted exercise program, beginning in the pre-season and containing at least three exercise types, may be beneficial in reducing ACL injury risk.

## 1. Introduction

Anterior cruciate ligament (ACL) injuries account for a large proportion (20.5%) of knee injuries among youth athletes, often leading to devastating consequences [1]. In addition to the significant financial burden associated with surgical intervention and extensive rehabilitation [2], ACL injuries result in a personal cost to athletes, with time lost from the sport, psychological effects, and consequences to long-term health and wellbeing [3,4]. While the ACL is the most commonly damaged ligament, ACL injuries typically involve damage to surrounding tissues as well [5]. Such injuries result in an increased risk of subsequent knee injury and chronic knee problems, including early onset osteoarthritis [6]. In fact, one study demonstrated radiographic evidence of knee osteoarthritis among 42% of female soccer players following ACL reconstruction within 10 years of the injury [7]. Thus, primary prevention is an effective way to reduce the adverse physical, psychological, and financial outcomes of this traumatic injury [8].

Approximately 70% of all ACL tears occur with a non-contact mechanism [9,10], suggesting that a significant number of these tears might be avoided by intervening with prevention programs. Several ACL injury prevention programs focusing on the reduction in non-contact ACL injuries have been developed to target high-risk populations, such as young female athletes [11]. Epidemiological research has demonstrated that female athletes have a two to eight times higher risk of ACL injury compared to their male counterparts [1,12,13]. The increased injury risk, coupled with increased participation in sports by young females has led to a significant volume of interventional research focusing on female participants [12,14]. Previous research has indicated that sex-specific anatomical, hormonal, neuromuscular, and biomechanical factors predispose females to an increased risk of non-contact ACL injuries [15]. Because anatomical and hormonal factors are non-modifiable, most prevention programs have focused on modifiable neuromuscular and biomechanical risk factors. Specific neuromuscular and biomechanical risk factors for young females include increased knee abduction angles, reduced knee and hip flexion, decreased core strength and proprioception, decreased hamstring strength relative to quadriceps, increased hip internal rotation, and tibial external rotation with or without pronation of the foot [15].

ACL injury prevention programs vary in their framework and implementation methods. These prevention programs have emphasized neuromuscular and biomechanical training to promote muscular support around the knee and alter movement patterns [16]. Combinations of warm-up, balance training, stretching, strength training, and plyometrics, as well as exercises that improve agility and increase awareness of high-risk movements, have been included in these interventions [16]. While prophylactic neuromuscular training was shown to be beneficial, there remains a large number needed to treat (108) to prevent one ACL injury [17]. This indicates that a thorough investigation of the most effective components to include in ACL injury prevention programs and screening for high-risk athletes is warranted. An examination of differences in the delivery of prevention interventions, particularly the frequency, duration, and feasibility of the programs, as well as timing within the training year, needs to be considered. Moreover, the age of athletes participating in the prevention programs should be considered, as female athletes under 19 years of age appear to have a greater prophylactic effect from training compared to older adults [18], indicating the importance of initiating preventative programs during early adolescence.

Assisting health professionals and physicians to stay current on the most recent evidence-based findings, there has been a large number of systematic reviews and meta-analyses conducted that examine ACL injury prevention programs. As this literature increases in volume, it can be difficult for clinicians to stay current on this topic. Therefore, umbrella reviews, otherwise known as “overviews”, “review of reviews”, and “meta-reviews”, have increased in popularity as a form of evidence synthesis to provide user-friendly summaries for healthcare providers [19]. To our knowledge, there have been no umbrella reviews examining the effectiveness of ACL injury prevention training programs, despite the considerable number of systematic reviews and/or meta-analyses. There has been only one recent meta-analysis of meta-analyses examining the effectiveness of ACL injury prevention training programs covering the period 2006–2015 [11]. While there have been many systematic reviews on the topic, they did not meet the inclusion criteria for the recent study [11]. Thus, we aimed to provide a more recent meta-analysis and synthesis of the literature, including analyses from systematic reviews, examining the effectiveness of non-contact ACL injury prevention programs among young female athletes for the period from database inception to May 2021.

The purposes of this study were to (1) perform an umbrella review in order to evaluate the effectiveness of ACL injury prevention programs to reduce the risk of non-contact ACL injuries among young female athletes under 19 years of age, (2) determine the specific effective components to include in non-contact ACL injury prevention programs, and (3) provide clinical recommendations for family physicians and general practitioners to aid in prevention and counseling.

## 2. Materials and Methods

The review protocol was registered in PROSPERO (ID: CRD42021253575). The addition of a secondary search for primary literature was an amendment to the registered protocol as studies from 2014 to 2021 were not captured in the most recent review that met our inclusion criteria. The review was conducted in accordance with the Preferred Reporting Items for Systematic Reviews and Meta-Analyses (PRISMA) guidelines [20].

### 2.1. Study Types

Systematic reviews and/or meta-analyses of randomized control trials or prospective cohort studies that evaluated the effectiveness of ACL injury prevention programs were included in this umbrella review. We limited our review to the examination of previous studies that included randomized control trials and prospective cohort studies due to the inherent limitations of cross-sectional and case–control study designs. Reviews that included randomized control trials, prospective cohort studies, and other types of primary studies were included; however, other study designs were excluded from our analysis.

In an attempt to ensure this umbrella review included all relevant primary literature up to the present year, we performed another search for primary literature that was published after the most recent systematic review and/or meta-analysis related to our research question. The study design for the secondary search was also restricted to randomized control trials and prospective cohort studies for the same reason noted above.

### 2.2. Participants

Studies that included female athletes under 19 years old participating in any sport were included in this umbrella review. Reviews that included primary literature containing male athletes only and/or athletes over the age of 19 years were included in our review; however, the primary study was omitted from our final analysis.

### 2.3. Interventions

Studies that examined interventions aimed at preventing primary non-contact ACL injuries that compared an intervention group with a control group that did not receive any intervention were included in this umbrella review.

### 2.4. Outcome Measures

Studies that examined changes in non-contact ACL injuries (e.g., incidence rate, odds ratio, risk ratio, or relative risk reduction) following the intervention were included in this umbrella review. Primary literature that measured the number of non-contact ACL injuries in both control and intervention groups was included in the meta-analysis if athlete-exposure data were available or incidence rates (per athlete exposures) were provided.

### 2.5. Exclusion Criteria

Reviews were excluded based on the following exclusion criteria: (1) narrative reviews without a search algorithm or that failed to describe how studies were selected for the review, (2) only adults or male athletes were included as participants, (3) reviews that examined rehabilitation intervention post ACL injury or post ACL surgical intervention, (4) reviews that examined general or sports injury prevention programs that were not specific to ACL injury prevention, and (5) reviews that failed to differentiate contact ACL injury from non-contact ACL injury in the results and analyses. Eligible reviews written in a language other than English were carried forward until the full-text review process and subsequently excluded due to the lack of feasibility for translation.

Primary literature found during the secondary search was excluded based on the following criteria: (1) only adults or male athletes were included as participants, (2) the study examined a rehabilitation intervention post ACL injury or post ACL surgical intervention, (3) the study examined a general or sports injury prevention programs that were not specific to ACL injury prevention, and (4) the study failed to report the mechanisms of ACL injury in the results. Authors of the primary studies that fulfilled all of the criteria except for reporting the mechanism of ACL injuries were contacted for further information and were included if the details could be obtained.

### 2.6. Sources of Information

The following electronic databases were searched for the umbrella review from the inception of the database to 13 May 2021: Medline, Embase, and Cochrane Database of Systematic Reviews through the OVID platform; Cumulative Index to Nursing and Allied Health Literature (CINAHL), and SPORTDiscus through the EBSCOhost platform; and PEDro, Web of Science Core Collection, and Epistemonikos. In order to avoid publication bias favoring positive results, the following electronic databases were searched for grey literature: ProQuest Dissertations and Theses Global, CPG Infobase, BC Guidelines and Protocols, and TRIP. All databases were searched using a systematic review filter developed by Boynton et al. [21] due to its high sensitivity score for systematic reviews and meta-analyses [22]. The reference lists of all included studies were manually searched to locate any relevant articles that may have been missed. All searches were conducted with the guidance of a biomedical librarian. The detailed search strategy for each database is listed in Appendix A.

The following electronic databases were explored for the secondary literature search: Medline, Embase, CINAHL, and SPORTDiscus through the EBSCOhost platform. The searches were conducted on 27 September 2021 and were limited to 2014 and beyond, as the most recent meta-analysis included in our umbrella review ran their searches from the inception of the database to 2014 [23]. We limited our search to the four most commonly searched databases across all systematic reviews and meta-analyses, therefore we are confident we have the most recently published literature not included in previous reviews related to our research question. The reference lists of all included studies were manually searched to locate any relevant articles that may have been missed. The detailed search strategy is the same as listed in Appendix A, with the exception of the systematic review filter developed by Boynton et al. [21].

### 2.7. Selection of Studies

The titles and abstracts of potentially relevant studies were independently screened by two authors (A.T.M. and B.G.) and excluded if they did not meet the selection criteria. Any disagreements were resolved with a discussion until a consensus was reached. In any case where a disagreement could not be resolved with a discussion, disagreements were arbitrated by a third author (I.P.). Following the title and abstract screening, two authors (A.T.M. and B.G. for the initial search, A.T.M. and V.L. for the secondary search) independently reviewed the full-texts of potentially relevant articles against the inclusion and exclusion criteria. The same process for resolving disagreements applied and the specific reasons for exclusion were documented. Inter-rater agreement was calculated for both screening procedures using a Cohen’s kappa score with values interpreted as ≤0 indicating no agreement, 0.01–0.20 indicating slight agreement, 0.21–0.40 indicating fair agreement, 0.41–0.60 as moderate agreement, 0.61–0.80 as substantial agreement, and 0.81–1.00 as almost perfect agreement. The process of study selection for both literature searches was identical.

### 2.8. Data Extraction and Management

Two authors (A.T.M. and B.G. or V.L.) independently extracted data from all included articles. A data extraction form was designed using the Covidence systematic review software (Veritas Health Innovation, Melbourne, Australia, available at www.covidence.org (accessed on 4 May 2021)) in order to standardize the process between both reviewers. The following data were collected from the reviews: general review information (title, authors, corresponding author details, publication journal and year, study setting, study funding sources, and conflicts of interest), characteristics of the review (study objective, study design, inclusion and exclusion criteria, databases searched, and date of the last literature search), characteristics of the included studies (title, author, publication year, study design, sample size and distribution between the intervention and control groups, sex of the participants, age of the participants, and sports included), intervention details of included studies (program name, exercise types, frequency, duration, and length of intervention, total number of training sessions, training season, equipment required, supervision of intervention, and compliance rate), outcome measures of included studies (number of non-contact ACL injuries for the control and intervention groups, athlete exposure data, and ACL injury rates with 95% confidence interval), results of the review (total number of primary studies included, total number of participants, heterogeneity analysis between primary studies if applicable, and pooled results if applicable), and the findings/conclusions of the review.

The following data were collected from the primary literature: general information (title, authors, corresponding author details, publication journal and year, study setting, sources of funding, and conflicts of interest), characteristics of the included studies (title, author, publication year, study design, sample size and distribution between the intervention and control groups, sex of the participants, age of the participants, and sports included), intervention details of included studies (program name, exercise types, frequency, duration, and length of intervention, total number of training sessions, training season, equipment required, supervision of intervention, and compliance rate), outcome measures of included studies (number of non-contact ACL injuries for the control and intervention groups, athlete exposure data, and incidence rates), and the findings/conclusions of the study.

We defined six exercise types: warm-up, plyometrics, strength, agility, balance, and stretching. Warm-up included aerobic exercises, muscle activation, or unspecified basic warm-up, such as jogging. Plyometrics included jumping and rebound exercises. Strength comprised eccentric or concentric strength training, weight training, core stability, or power training. Agility involved sport cord drills, sport-specific drills, or running techniques. Balance included proprioception, neuromuscular training with wobble boards or balance mats, dynamic stability, body control, or one-leg coordination. Stretching involved any flexibility exercises. These exercise type definitions were adopted from an umbrella review examining the effectiveness of exercise combinations within lower extremity injury prevention programs [24].

### 2.9. Risk of Bias Assessment in Included Studies

Two review authors (A.T.M. and B.G.) independently assessed the risk of bias in all the included reviews. The quality of both systematic reviews and/or meta-analyses was assessed using a Measurement Tool to Assess Systematic Reviews 2 (AMSTAR 2). Disagreements were resolved with a discussion and, if necessary, a consensus was reached with the involvement of a third reviewer (I.P.). The AMSTAR 2 quality assessment tool defines seven critical domains: protocol registered before the commencement of the review, adequate literature search, justification for excluding individual studies, risk of bias from individual studies included in the review, appropriate meta-analytical methods, considerations of risk of bias when interpreting results of the review, and assessment of the prevention and likely impact of publication bias [25]. The rating of overall confidence in the results of the review included critically low (more than one critical flaw with or without non-critical weaknesses), low (one critical flaw with or without non-critical weaknesses), moderate (more than one non-critical weakness), and high (no or one non-critical weakness) [25].

Two review authors (A.T.M. and V.L.) independently assessed the risk of bias in all included primary literature. A revised Cochrane Risk-of-Bias Tool for Randomized Trials (RoB 2) was used to assess the quality of the primary literature. Disagreements were resolved with a discussion and, if necessary, a consensus was reached with the involvement of a third reviewer (I.P.). The most appropriate RoB 2 tool was used based on the study design (i.e., RoB 2 for individually randomized, parallel-group trials or RoB 2 for cluster-randomized trials). The RoB 2 assessment tool is structured into five domains of bias [26]. Each domain contains a series of questions aimed to extract qualities of the trial that are relevant to the risk of bias [26]. A proposed judgment of either low or high risk of bias, or some concerns, for each domain and the study overall was generated using an algorithm based on the answers to the questions within each domain [26].

### 2.10. Data Synthesis

A qualitative assessment of all the included reviews was performed. When there were any discrepancies in the extracted data across reviews, we referred to the primary literature to ensure the correct information was extracted. Many of the meta-analyses included the same primary studies in their analysis, making it inappropriate to perform a meta-analysis of meta-analyses due to the potential overrepresentation of studies with earlier publication dates. Instead, we performed an updated meta-analysis of the primary literature, including the studies incorporated in past meta-analyses, as well as any new studies since the last meta-analysis was performed.

The incidence rate ratio (IRR) was used to measure the effects of prevention programs on ACL injury, comparing the ACL incidence rate in the intervention group (numerator) with the incidence rate in the control group (denominator). The IRRs were calculated from the number of non-contact ACL injuries and athlete exposure (AE) events (i.e., game or practice). In the cases where exposure events were not provided, the IRR was obtained directly from the study. AE data were reported in hours for some studies [27,28,29,30,31]. To standardize the AE data, AE hours were converted to AE events based on the calculation of two exposure hours equals one AE event, as used in previous studies [32,33]. Incidence rates were estimated as the number of non-contact ACL injuries per 1000 AE events.

We performed an inverse variance weighted meta-analysis to estimate the overall pooled effect using a random-effects model. A forest plot was used to graphically summarize the IRR across studies and the pooled effect, along with the 95% confidence intervals. We assessed the publication bias and small sample bias graphically using a funnel plot. We assessed the heterogeneity between studies using Cochran’s Q and *I*^2^ statistics (Vienna, Austria).

## 3. Results

### 3.1. Literature Search Results

#### 3.1.1. Reviews

**Figure 1:**
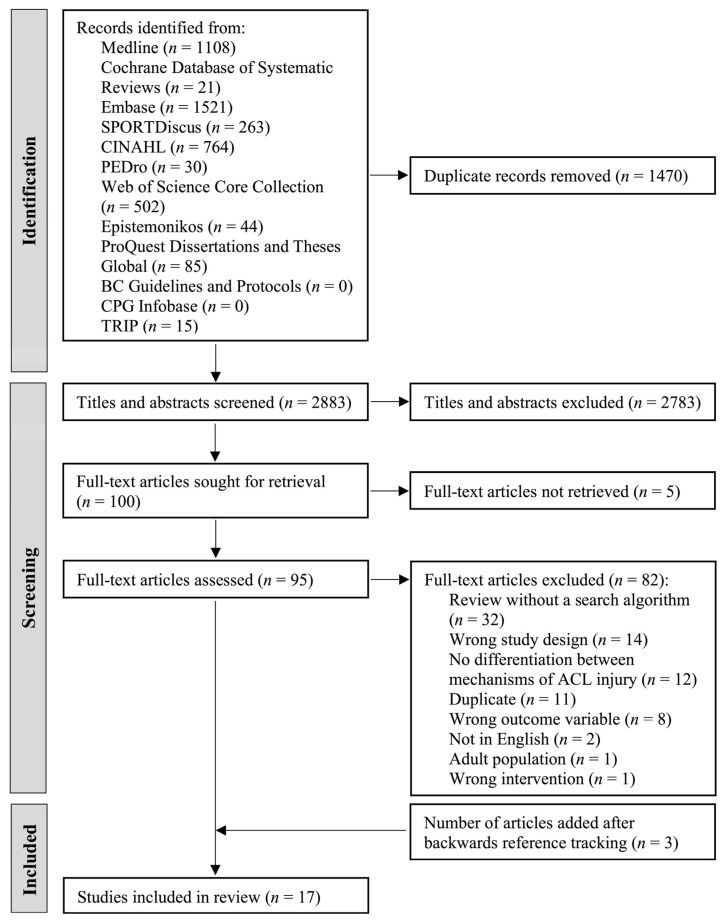
PRISMA flow diagram of the literature search for review articles.

The search resulted in 4353 records being found. After the removal of duplicates, 2883 remained. There were 54 conflicts for the title and abstract screening (κ = 0.742, *p* < 0.0005), indicating substantial agreement between reviewers. After screening titles and abstracts, 100 studies were moved to the full-text review. Of the 100 papers, five articles could not be retrieved. The 95 articles assessed represented 84 unique studies. There were seven disagreements between reviewers (κ = 0.724, *p* < 0.001), indicating substantial agreement for the full-text review. In cases where multiple papers described the same study, only the most comprehensive publication was retained for further analysis. Of the articles reviewed, 14 articles met the inclusion criteria and 81 were excluded (Figure 1). Later, manual checking of the reference lists from relevant reviews yielded an additional three records, totaling 17 relevant papers. Although the review by Alentorn-Geli et al. [34] classified itself as a systematic review and was initially included during the full-text review, it was in fact a narrative review and subsequently excluded prior to data extraction. The reasons for the exclusion of articles in the full-text review are listed in Appendix B, Table A1.

#### 3.1.2. Primary Literature

**Figure 2:**
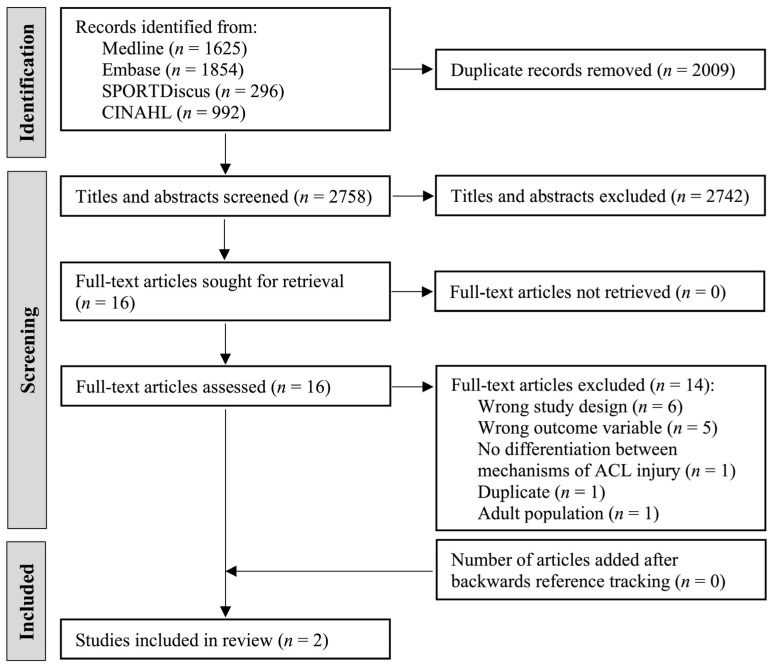
PRISMA flow diagram of the literature search for primary articles.

The search yielded 4767 records. After the removal of duplicates, 2758 records remained. After screening titles and abstracts, 16 studies were moved to the full-text review. There were two conflicts between reviewers for the title and abstract screening (κ = 0.937, *p* < 0.0005), indicating almost perfect agreement. Of the 16 papers, two met the inclusion criteria and 14 were excluded (Figure 2). There was one conflict between reviewers for the full-text review (κ = 0.765, *p* < 0.002), indicating substantial agreement. The reasons for the exclusion of articles in the full-text review are listed in Appendix B, Table A2.

### 3.2. Study Characteristics

#### 3.2.1. Reviews

There were 16 reviews included in our final analysis, published between 2005 and 2015. Eight of the reviews were systematic reviews [35,36,37,38,39,40,41,42], two were meta-analyses [23,43], and six were systematic reviews with meta-analyses [17,32,33,44,45,46]. Eleven of the reviews were conducted in the United States of America (USA) [17,23,32,33,35,36,37,40,42,44,46], another was conducted in Austria and USA [45], and one review each was conducted in Greece [41], Serbia [38], South Korea [43], and England [39]. Characteristics of the included systematic reviews are summarized in Table 1. Characteristics of the included meta-analyses and systematic reviews with meta-analyses are summarized in Table 2. Although Gagnier et al. reported that the outcome variable of their review was non-contact ACL injuries, they included primary studies with data on all ACL injuries and did not specify which studies recorded non-contact ACL injuries [46]. Subsequently, Gagnier et al. was removed from the qualitative analysis.

#### 3.2.2. Primary Literature

Eight unique primary studies related to our research question, published between 1999 and 2012, were included across the 15 reviews (Table 3). An additional two primary studies, published between 2014 and 2021 were found in the secondary search. Across the 10 primary articles, there were six randomized control trials [27,28,30,31,47,48] and four prospective cohort studies [29,49,50,51]. Four studies were conducted in the USA [47,49,50,51], two in Norway [27,28], two in Sweden [29,30], one in Germany [31], and one in Thailand [48]. Characteristics of the included primary studies are summarized in Table 4. Intervention details of the included primary studies are summarized in Table 5.

### 3.3. Risk of Bias in Included Studies

#### 3.3.1. Reviews

**Figure 3:**
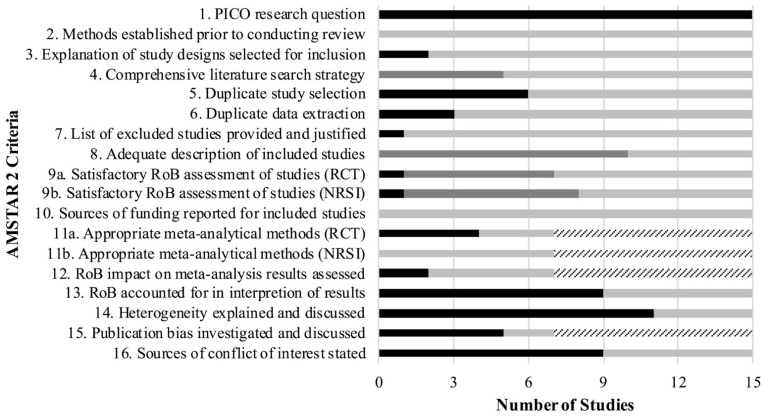
Quality assessment of the reviews using the AMSTAR 2 tool. RCT: randomized control trial; NSRI: non-randomized studies of interventions.

Of the 15 systematic reviews and meta-analyses, only one review was rated as low [39], while the other 14 reviews were rated as critically low using the AMSTAR 2 quality assessment tool [17,23,32,33,35,36,37,38,40,41,42,43,44,45] (Figure 3).

#### 3.3.2. Primary Literature

Of the two additional primary studies included in the meta-analysis, both studies had some concerns overall with regards to the risk of bias using the RoB 2.0 criteria. Achenbach et al. [31] had a low risk of bias in the following categories: timing of participant identification or recruitment of participants, missing outcome data, and measurement of the outcome; however, there were some concerns about the risk of bias in the following categories: randomization process, deviations from the intended interventions, and selection of the reported result. Yarsiasat et al. [48] had a low risk of bias in the following categories: randomization process, deviations from the intended interventions, and missing outcome data; however, there were some concerns about the risk of bias in the following categories: measurement of the outcome and selection of the reported result.

### 3.4. Effectiveness of ACL Injury Prevention Programs

Of the seven meta-analyses included in this review, only five studies conducted a subgroup analysis for non-contact ACL injury. All five meta-analyses showed a significant reduction in non-contact ACL injury risk following an ACL injury prevention program (Table 2). Two of the reviews calculated odds ratios [32,43], one review each calculated a risk ratio [45], an incidence rate reduction related to AEs [23], and a relative risk reduction with a number needed to treat value [17]. Taken together, these meta-analyses demonstrated a 49% to 73% reduction in non-contact ACL injury risk following an injury prevention program (Table 2).

The conclusions from 14 of the 15 included reviews aligned with the interpretation of the pooled results, indicating that there is a substantial beneficial effect of ACL injury prevention programs in reducing non-contact ACL injuries. One systematic review concluded that injury rates between the intervention and control groups of the included studies were not significantly different in most instances [41].

**Figure 4:**
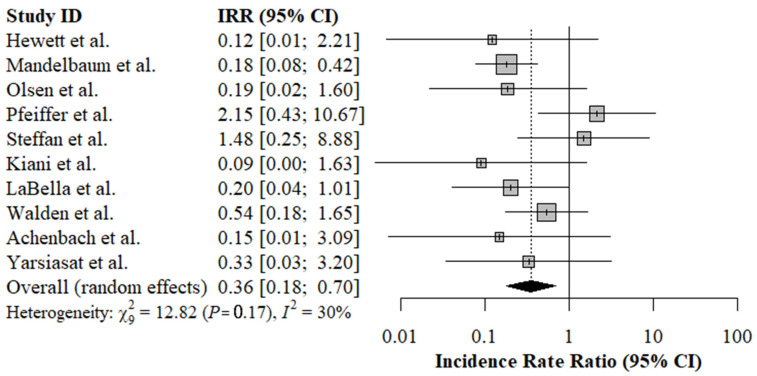
Main effect meta-analysis: random-effects, inverse variance weighted model. IRR: incidence rate ratio; CI: confidence interval.

**Figure 5:**
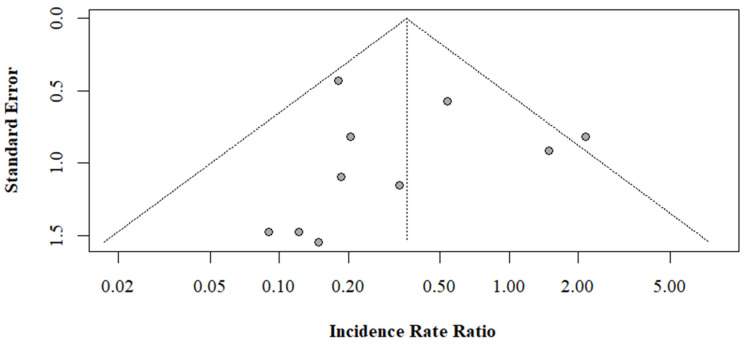
Funnel plot for the random-effect estimate.

Using the random-effects, inverse variance weighted model, we found that a pooled effect estimate for the IRR of primary literature was 0.36 (95% CI: 0.18, 0.70), indicating ACL injury prevention programs significantly reduced the rate of non-contact ACL injury by 64%. The results of the meta-analysis are presented graphically in a forest plot (Figure 4). The funnel plot of the 10 primary studies analyzed was fairly symmetric and within the bounds of the funnel, indicating low small-sample bias and low publication bias (Figure 5). The *I*^2^ value was 30% and the Q statistic results were not significant, indicating low heterogeneity between studies (Figure 5).

### 3.5. Specific Training Components

All 15 reviews explored the beneficial components of an ACL intervention program related to a reduction in all ACL injuries, with 11 reviews performing a qualitative analysis of the primary literature and four reviews performing a quantitative analysis using a meta-regression [23,32,45] or an odds ratio [43]. Three reviews did not come to any conclusion regarding which components were effective or ineffective in an ACL injury prevention program [37,39,45], and all studies were in agreement that the evidence from primary literature is insufficient to draw firm conclusions on which one component is superior.

Twelve reviews suggested that a multi-faceted training program was beneficial for reducing injury risk, with Herman et al. focusing on lower limb injury rates as opposed to ACL injury rates specifically [17,32,33,35,36,38,39,40,41,42,43,44]. Three reviews noted that neuromuscular training that only utilized one exercise type (specifically balance or plyometrics only), did not reduce ACL injuries in female athletes [17,38,42]. Through a qualitative analysis, reviews recommended the inclusion of three to five different exercise type combinations in an ACL prevention program. Eight reviews agreed that plyometrics should be included in a comprehensive ACL prevention program [33,35,36,38,40,41,42,44], six of which recommended including strength training as well [33,35,40,41,44]. Six, five, and two reviews also suggested including balance training [33,35,36,38,41,44], agility [36,38,40,41,44], and stretching exercises [40,44], respectively. In addition, six reviews stressed the importance of feedback or technique awareness as a key component of prevention programs [35,36,38,40,41,44].

From a quantitative perspective, Donnell-Fink et al. and Sadoghi et al. found no significant association between exercise components and ACL injury prevention, with Sadoghi et al. focusing on balance board and video-assistance only [23,45]. Yoo et al. found that plyometrics and strengthening exercises were essential components in prevention programs, while balancing exercises were not [43]. Taylor et al. found that greater emphasis on balance training and static stretching may be associated with an increase and decrease in ACL injury risk, respectively [32]. They also found no significant association between feedback or technique awareness on ACL injury [32].

### 3.6. Duration, Frequency, and Timing of Interventions

Stevenson et al. noted that it is unclear whether longer or more frequent training allows for the greatest benefit, as most training programs had comparable durations of training sessions and frequency with varying results [42]. Two studies agreed that interventions should be at least 6 weeks long, with one study indicating a frequency of at least more than one time per week [33], and the other suggesting beginning in the pre-season and continuing into the season with decreased frequency (1–2 times per week) [41]. Grindstaff et al. concluded that interventions should be 10 to 20 minutes long, at least three times per week in the pre-season, and then at least one time per week during the season [44]. Similarly, Sadoghi et al. suggested prevention programs should include at least 10 minutes of exercise three times per week [45]. With a meta-regression, Taylor et al. found no effect of total training time or session duration on the effectiveness of reducing injury risk [32].

Yoo et al. found training in both the pre-season and in-season was more effective than training in either season alone [43]. This conclusion was also drawn in two qualitative analyses [33,44]. Sadoghi et al. found a protective but not significant effect of training in the pre-season compared to in-season [45]. Donnell-Fink et al. found a protective but not significant effect of training in the pre-season or both seasons compared to in-season only [23]. The findings from Donnell-Fink et al. were echoed in the conclusions of Michaelidis et al. and Stevenson et al. [41,42].

## 4. Discussion

With the evidence available from 15 systematic reviews and/or meta-analyses, the primary consensus across the majority of reviews was that ACL injury prevention programs were effective in reducing non-contact ACL injuries in female athletes. Results from the current up-to-date meta-analysis echo this conclusion, as the estimated IRR was 0.36 (95% CI: 0.18–0.70), indicating neuromuscular prevention programs reduced the incidence of non-contact ACL injury in youth female athletes by 64%. Moreover, we found that although evidence was limited in supporting any one type of exercise component alone, a multi-faceted exercise program with at least three different exercise types and either technique awareness or feedback was beneficial in reducing ACL injury risk. The most frequently recommended exercise components to include in an ACL injury prevention program were plyometrics and strengthening exercises. While there was no conclusion to be drawn from the varying recommendations regarding the duration and frequency of ACL interventions, the most common consensus was to begin the intervention in the pre-season and continue into the season.

### 4.1. Effectiveness of ACL Injury Prevention Programs

Neuromuscular injury prevention programs were designed to target non-contact ACL injury [17,33,52,53]. The findings from our meta-analysis aligned with the most recent meta-analysis of meta-analyses, which found a 67% reduction in the risk of non-contact ACL injuries in female athletes [11]. The effectiveness of ACL prevention programs was shown to be reduced when considering overall ACL injuries compared to strictly non-contact ACL injuries [17,24,43]. Therefore, it is important for reviews and primary studies to make a distinction between contact and non-contact mechanisms of injury when examining the true effectiveness of prevention programs overall and across subgroups.

Compliance is an important factor to consider when examining the benefits of ACL injury prevention programs. Due to the limited number of reviews extracting and reporting compliance data from the primary literature and the varying ways of calculating compliance across the reviews, we were unable to synthesize compliance results. However, some reviews noted the major limitation to robustly assessing compliance was the lack of complete data and standardization of compliance definitions across primary studies [33,42,44]. Nevertheless, a previous meta-analysis showed that greater levels of compliance were associated with lower rates of ACL injuries [54].

### 4.2. Intervention Components

Multi-component exercise interventions were shown to be effective at reducing multiple lower extremity injury outcomes, including ACL injury [24]. Reviews recommended anywhere from three to five different exercise types be included within a prevention program. Plyometric and strengthening exercises were the two most common types of exercises recommended across reviews. While, the next most commonly recommended exercise type was balance training, some reviews that conducted meta-regressions found no association or an increased risk of ACL injury with greater emphasis on balance training [32,45]. Thus, it is unclear what impact balance training has on ACL injury risk. Michaelidis et al. specifically noted that sport-specific agility was key to include in prevention for soccer and handball players, while plyometrics was a vital component of prevention programs for basketball players [41]. This indicates that perhaps the importance of exercise type should be related to the high-risk movements within a specific sport rather than the implementation of one standardized program across all sports. Although not all reviews agreed on specific exercise combinations, at least three different exercise types were recommended. This aligns with the recent National Athletic Trainers Association Position (NATA) statement, which recommended that injury prevention programs include at least three exercise types, as well as feedback, to reduce the risk of non-contact ACL injury [53].

The recent NATA statement also recommended that multi-component training programs should be performed at least two to three times per week throughout the pre-season and season [53]. Our findings aligned with the training season recommendations from NATA. The data on the frequency of training was inconsistent across reviews and, therefore, inconclusive. However, another systematic review and meta-analysis specifically examining dosage effects of neuromuscular training interventions on the reduction of all ACL injuries found a more significant prophylactic effect in longer-duration, multi-frequency programs compared to short-duration, single-session programs [55].

### 4.3. Quality Assessment

To ensure that the highest quality of evidence available was included in our review, we used the AMSTAR 2 quality assessment tool. Using this tool, only one review was rated as low, while the rest were rated as critically low. While this was a surprising result, it emphasizes the importance of transparency when reporting the review process. With this in mind, the AMSTAR 2 tool was developed in 2017, two years after the last review was published. With the ever-evolving changes in methodological reporting of systematic reviews and meta-analyses, it is likely that the reporting requirements were different at the time of publication for these reviews. Therefore, the findings from these reviews were still considered important in this umbrella review. In the two additional primary studies, there were some concerns related to the risk of bias. Similar to the reviews, this was largely due to the lack of information to make a definitive decision about items on the RoB 2 tool, once again highlighting the importance of transparency in reporting study methodology.

### 4.4. Strengths and Limitations

There were several strengths of this umbrella review. This was the first umbrella review to combine data from both meta-analyses and qualitative analyses from systematic reviews. An updated meta-analysis on the effectiveness of ACL injury prevention programs for reducing non-contact ACL injury risk was also performed. Moreover, this review followed strict inclusion criteria related to the mechanisms of ACL injuries and did not include reviews that failed to differentiate between contact and non-contact ACL injuries in their analyses. This was done in order to get a better understanding of the true impact of injury prevention programs on ACL injury risk. Finally, this review provides healthcare providers and general practitioners with a succinct and up-to-date overview of the literature for easy access.

This umbrella review was not without its limitations. Our findings were limited to the reviews on which they were based. Given that none of the reviews examined the long-term effects of the prevention programs, we cannot say with certainty that the decreased risk of ACL injury is sustained in the long term. Another potential limitation is the difference in classification of exercise types across reviews or the failure to standardize the definition of exercise types. For example, the study by Kiani et al. classified jump training as balance, whereas it was interpreted as plyometrics, power, or jump and landing exercises in other studies and reviews [29]. The failure to operationalize the definition of exercise types may have led to misclassification within reviews. This increased the risk of reporting bias. However, to minimize this risk, we referred to the primary literature whenever there was a discrepancy to ensure that there was one standardized definition across our umbrella review.

### 4.5. Recommendations and Future Directions

For general practitioners and family physicians, we recommend encouraging all female athletes who engage in high-risk sports involving jumping, landing, and cutting to engage in an injury prevention program to decrease their risk of non-contact ACL injury. Moreover, the intervention program should include plyometrics and strength components, as well as at least one other exercise type (agility, balance, or stretching). Emphasis on technique or feedback should be provided to athletes through the program. Finally, athletes should begin these programs in the pre-season and continue them throughout the season.

## 5. Conclusions

ACL injury prevention programs were effective at reducing non-contact ACL injuries in youth female athletes. A multi-faceted exercise program, beginning in the preseason and continuing throughout the season appears to be beneficial for reducing ACL injury risk. Intervention programs should include at least three different exercise types, especially plyometric and strengthening exercises, and include feedback or emphasis on technique. Individual exercises should be related to the high-risk movements within each specific sport.

Compliance is another crucial aspect of the success of an ACL injury prevention program. An emphasis should be placed on encouraging athletes to complete all training sessions to ensure adequate neuromuscular and biomechanical adaptations are achieved. Incorporating a prevention program within a warm-up may improve adherence to the training.

## Figures and Tables

**Table 1:** Characteristics of the included systematic reviews.

Study ID	Databases Searched	Date of Last Search	Total Number of Studies * (Non-Contact)	Total Number of Participants * (Non-Contact)	Study Funding
Hewett et al. [35]	Medline, CINAHL	NR	5 (3)	8764 (8324)	NIH
Padua et al. [36]	PubMed	NR	5 (2)	8850 (6532)	NR
Noyes et al. [37]	PubMed, CINAHL, Science Direct	August 2011	6 (4)	13,067 (8400)	NR
Stojanovic et al. [38]	Medline	June 2011	9 (5)	13,884 ^†^ (NR)	NR
Herman et al. [39]	PubMed, Embase, SPORTDiscus, PEDro, Google Scholar, ISI Web of Knowledge, Scirus	January 2012	6 (3)	8999 (4034)	NR
Noyes et al. [40]	Medline, CCRCT	May 2013	9 ^#^ (9)	19,493	NR
Michaelidis et al. [41]	PubMed, CINAHL, CCRCT, Science Direct, SPORTDiscus, PEDro, SCOPUS	September 2012	13 (11)	22,052 (21,612)	None
Stevenson et al. [42]	Medline, CINAHL, CCRCT	2011	10 (5)	15,512 (5771)	NR

CINAHL: Cumulative Index to Nursing and Allied Health Literature; CCRCT: Cochrane Central Register of Controlled Trials; NR: Not reported; NIH: National Institutes of Health grant; *: related to all ACL injuries; ^#^: includes one unpublished update; ^†^: included male participants.

**Table 2:** Characteristics of the included systematic reviews and meta-analyses.

Study ID	Databases Searched	Date of Last Search	Total Number of Primary Studies * (Non-Contact)	Total Number of Participants (Non-Contact)	Meta-Analysis Results ^†^	Reduction in Non-Contact ACL Risk	Study Funding Sources
Hewett et al. [33]	Medline, CINAHL	2004	6 (3)	9040 (8324)	Not conducted	-	NIH
Grindstaff et al. [44]	PubMed, Medline, CINAHL, SPORTDiscus, Web of Science	October 2005	5 (5)	11,026	Not conducted	-	NR
Yoo et al. [43] *	Medline, CCRCT	June 2007	7 (4)	10,618 (8247)	OR = 0.36 (0.23–0.54)	64%	NR
Sadoghi et al. [45]	PubMed, Medline, CINAHL, CCRCT, Embase	December 2010	8 (6)	10,618 (9982)	RR = 0.48 (0.26–0.89)	62%	None
Sugimoto et al. [17]	PubMed, Medline, CINAHL, SPORTDiscus	January 2012	12 (10)	18,523 (18,083)	RRR = 73.4% (63–81)	73.4%	NIH
					NNT = 108 (86–150)		
Gagnier et al. [46] ^^^	Medline, CINAHL, CCRCT, Embase, SPORTDiscus, HTA	July 2011	14 (NR)	~27,136 (NR)	Not conducted	-	U of M Bone & Joint Injury Prevention & Rehabilitation Center
Taylor et al. [32]	PubMed, Medline, CINAHL, CCRCT, SPORTDiscus	July 2012	13 (9)	24,188 (19,891)	OR = 0.38 (0.22–0.64)	62%	None
Donnell-Fink et al. [23] *	PubMed, Medline, CINAHL, CCRCT, Embase, Web of Science	December 2014	14 (9)	20,132 (15,235) ^#^	IRR = 0.51 (0.30–0.88)	48.7%	NIH, NIAMSD

CCRCT: Cochrane Central Register of Controlled Trials; CINAHL: Cumulative Index to Nursing and Allied Health Literature; HTA: Health Technology Assessment; OR: odds ratio; RR: risk ratio; RRR: relative risk reduction; NNT: number needed to treat; IRR: incidence rate ratio; NR: not reported; NIH: National Institutes of Health grant; U of M: University of Michigan; NIAMSD: National Institute of Arthritis and Musculoskeletal and Skin Diseases grant; *: meta-analysis only; ^#^: not all studies included in the meta-analysis and included male participants; ^†^: pooled results for non-contact ACL injury only, not restricted to youth; ^^^: removed from the final analysis due to failure to differentiate the mechanism of ACL injury.

**Table 3:** Primary studies included in the systematic reviews and meta-analyses.

Systematic Review and/or Meta-Analysis	Primary Study							
	Hewett et al. [49]	Mandelbaum et al. [50]	Olsen et al. [27]	Pfeiffer et al. [51]	Steffen et al. [28]	Kiani et al. [29]	LaBella et al. [47]	Walden et al. [30]
Hewett et al. [35] *	X	X						
Padua et al. [36] *	X	X	X					
Hewett et al. [33]	X	X						
Grindstaff et al. [44]	X	X	X					
Yoo et al. [43] ^#^	X	X		X				
Noyes et al. [37] *	X	X		X	X			
Sadoghi et al. [45]	X	X		X				
Stojanovic et al. [38] *	X	X		X				
Herman et al. [39] *		X		X	X	X	X	
Sugimoto et al. [17]	X	X	X	X	X	X	X	
Noyes et al. [40] *	X	X	X	X	X	X	X	X
Michaelidis et al. [41] *	X	X	X	X	X	X		X
Taylor et al. [32]	X	X	X	X	X	X	X	X
Stevenson et al. [42] *	X	X		X	X	X		
Donnell-Fink et al. [23] ^#^	X	X	X	X	X	X	X	X

*: systematic review only; ^#^: meta-analysis only.

**Table 4:** Characteristics and injury results of all primary studies.

Study ID	Study Design	Number of Participants	Age (Years)	Sports	Number of Injuries	ACL Injury Incidence *
Hewett et al. [49]	Prospective cohort study	C: 463	14–18	Soccer, basketball, volleyball	C: 5	0.22
		I: 366			I: 0	0.00
Mandelbaum et al. [50]	Prospective cohort study	C: 3818	14–18	Soccer	C: 67	0.49
		I: 1885			I: 6	0.09
Olsen et al. [27]	Randomized controlled trial	C: 778	15–17	Handball	C: 5	0.11
		I: 808			I: 1	0.02
Pfeiffer et al. [51]	Prospective cohort study	C: 862	14–18	Soccer, basketball, volleyball	C: 3	0.08
		I: 577			I: 3	0.17
Steffen et al. [28]	Randomized controlled trial	C: 947	13–17	Soccer	C: 2	0.06
		I: 1073			I: 3	0.09
Kiani et al. [29]	Prospective cohort study	C: 729	13–19	Soccer	C: 5	0.15
		I: 777			I: 0	0.00
LaBella et al. [47]	Randomized controlled trial	C: 755	16.2 ± 1.1	Soccer, basketball	C: 6	0.48
		I: 737	16.2 ± 1.5		I: 2	0.10
Walden et al. [30]	Randomized controlled trial	C: 2085	12–17	Soccer	C: 8	0.12
		I: 2479			I: 5	0.07
Achenbach et al. [31]	Randomized controlled trial	C: 215	15.1 ± 1.0	Handball	C: 2	0.32
		I: 259	14.9 ± 0.9		I: 0	0.00
Yarsiasat et al. [48]	Randomized controlled trial	C: 26	14–19	Sepak takraw	C: 3	0.05
		I: 26			I: 1	0.02

*: ACL injury incidence per 1000 athlete-exposure events.

**Table 5:** Summary of intervention details for the primary studies.

Study ID	Program Name	Exercise Types Included						Feedback	Duration (min.)	Frequency (d/wk.)	Length of Program	Training Season	Equipment
		Warm-Up	Plyometrics	Strength	Agility	Balance	Stretching						
Hewett et al. [49]	Sportsmetrics		X *	X			X		60–90	3	6 weeks	PS	Gymnastic mat, cones
Mandelbaum et al. [50]	PEP	X	X *	X	X		X		20	2–3	12 weeks for 3 seasons	S	Cones
Olsen et al. [27]	OLSEN	X	X *	X	X	X		X	15–20	15 sessions then 1	8 months	S	Wobble board, balance mat
Pfeiffer et al. [51]	KLIPP		X		X *				20	2	2 seasons	S	None
Steffen et al. [28]	The “11”	X	X	X	X	X		X	20	15 sessions then 1	8 months	PS, S	Balance mat
Kiani et al. [29]	HPT	X	X *	X	X				20–25	2 (PS), 1 (S)	9 months	PS, S	None
LaBella et al. [47]	KIPP	X	X	X	X	X		X	20	2–5 ^#^	13 ± 2.5 weeks	S	None
Walden et al. [30]	Walden		X	X		X		X	15	2	7 months	S	None
Achenbach et al. [31]	HSIPP		X	X		X			15	2–3 (PS), 1 (S)	10–12 weeks + season	PS, S	None
Yarsiasat et al. [48]	PEP	X	X	X	X		X		20	3	8 weeks	Unsure	Cones

PEP: Prevent Injury and Enhance Performance; KLIPP: Knee Ligament Injury Preventive Program; HPT: HarmoKnee Preventative Training Program; KIPP: Knee Injury Prevention program; HSIPP: Handball-Specific Injury Prevention Program; PS: pre-season; S: in-season; *: emphasis on technique; ^#^: before team practices and an abbreviated version with dynamic motion exercises only before games.

## Data Availability

No new data were created or analyzed in this study. Data sharing is not applicable to this article.

## Appendix A

### Appendix A.1. Medline Search Terms

1. Anterior Cruciate Ligament Injuries/
2. (Anterior Cruciate Ligament adj (Injur* or tear or rupture)).mp.
3. (ACL adj (Injur* or tear or rupture)).mp.
4. Knee Injuries/
5. Knee Injur*.mp.
6. 1 or 2 or 3 or 4 or 5
7. Athletic Injuries/pc [prevention & control]
8. (Prevent* or Intervention*).mp.
9. (Program* or Programme*).mp.
10. Exercise Therapy/or “Physical Education and Training”/or Physical Conditioning, Human/
11. Plyometric Exercise/or Muscle Stretching Exercises/or Resistance Training/or Warm-Up Exercise/
12. Training*.mp.
13. (Propriocept* or Plyometric* or Stretch* or Resistance* or Warm-up* or Neuromuscular* or Strength* or Condition* or Agility*).mp.
14. Education/
15. Educat*.mp.
16. Braces/
17. Brac*.mp.
18. 7 or 8 or 9 or 10 or 11 or 12 or 13 or 14 or 15 or 16 or 17
19. Adolescent/
20. (Adolesc* or Teen* or Youth* or Young*).mp.
21. Child/
22. Child*.mp.
23. Pediatrics/
24. P?ediatric*.mp.
25. 19 or 20 or 21 or 22 or 23 or 24
26. Female/
27. Female*.mp.
28. Athlete/
29. (Athlete* or Female Athlete*).mp.
30. 26 or 27 or 28 or 29
31. (meta or synthesis or literature).ab. or randomized.hw. or published.ab. or meta-analysis.pt. or systematic review.pt. or extraction.ab. or trials.hw. or controlled.hw. or search.ab. or MEDLINE.ab. or selection.ab. or sources.ab. or trials.ab. or review.ab. or review.pt. or articles.ab. or reviewed.ab. or english.ab. or language.ab.
32. 6 and 18 and 25 and 30
33. 31 and 32

### Appendix A.2. Embase Search Terms

1. anterior cruciate ligament injury/or anterior cruciate ligament rupture/
2. (Anterior Cruciate Ligament adj (Injur* or tear or rupture)).mp.
3. (ACL adj (Injur* or tear or rupture)).mp.
4. knee injury/
5. Knee Injur*.mp.
6. 1 or 2 or 3 or 4 or 5
7. sport injury/pc [prevention & control]
8. (Prevent* or Intervention*).mp.
9. (Program* or Programme*).mp.
10. kinesiotherapy/or leg exercise/or muscle training/or neuromuscular facilitation/or plyometrics/or stretching exercise/or muscle exercise/or resistance training/or “squatting (exercise)”/or warm up/or training/
11. Training*.mp.
12. (Propriocept* or Plyometric* or Stretch* or Resistance* or Warm-up* or Neuromuscular* or Strength* or Condition* or Agility*).mp.
13. education/
14. Educat*.mp.
15. brace/
16. Brac*.mp.
17. 7 or 8 or 9 or 10 or 11 or 12 or 13 or 14 or 15 or 16
18. adolescent/
19. (Adolesc* or Teen* or Youth* or Young*).mp.
20. child/
21. Child*.mp.
22. pediatrics/
23. P?ediatric*.mp.
24. 18 or 19 or 20 or 21 or 22 or 23
25. female/
26. Female*.mp.
27. athlete/
28. (Athlete* or Female Athlete*).mp.
29. 25 or 26 or 27 or 28
30. (meta or synthesis or literature).ab. or randomized.hw. or published.ab. or meta analysis/or “systematic review”/or extraction.ab. or trials.hw. or controlled.hw. or search.ab. or MEDLINE.ab. or selection.ab. or sources.ab. or trials.ab. or review.ab. or review.pt. or articles.ab. or reviewed.ab. or english.ab. or language.ab.
31. 6 and 17 and 24 and 29
32. 30 and 31

### Appendix A.3. Cochrane Database of Systematic Reviews Search Terms

1. (Anterior Cruciate Ligament adj (Injur* or tear or rupture)).mp.
2. (ACL adj (Injur* or tear or rupture)).mp.
3. Knee Injur*.mp.
4. 1 or 2 or 3
5. (Prevent* or Intervention*).mp.
6. (Program* or Programme*).mp.
7. Training*.mp.
8. (Propriocept* or Plyometric* or Stretch* or Resistance* or Warm-up* or Neuromuscular* or Strength* or Condition* or Agility*).mp.
9. Educat*.mp.
10. Brac*.mp.
11. 5 or 6 or 7 or 8 or 9 or 10
12. (Adolesc* or Teen* or Youth* or Young*).mp.
13. Child*.mp.
14. P?ediatric*.mp.
15. 12 or 13 or 14
16. Female*.mp.
17. (Athlete* or Female Athlete*).mp.
18. 16 or 17
19. 4 and 11 and 15 and 18

### Appendix A.4. SPORTDiscus Search Terms

S1. DE “ANTERIOR cruciate ligament injuries”
S2. (Anterior Cruciate Ligament N1 (Injur* or tear or rupture))
S3. (ACL N1 (Injur* or tear or rupture))
S4. DE “KNEE injuries”
S5. Knee Injur*
S6. S1 OR S2 OR S3 OR S4 OR S5
S7. DE “SPORTS injury prevention”
S8. Prevent* or Intervention*
S9. Program* or Programme*
S10. (DE “EXERCISE therapy” OR DE “EXERCISE therapy for children”) OR DE “PHYSICAL training & conditioning”
S11. DE “PLYOMETRICS” OR DE “STRETCH (Physiology)” OR DE “RESISTANCE training” OR DE “WARMUP” OR DE “STRENGTH training”
S12. Training*
S13. Propriocept* or Plyometric* or Stretch* or Resistance* or Warm-up* or Neuromuscular* or Strength* or Condition* or Agility*
S14. DE “EDUCATION”
S15. Educat*
S16. DE “SPORTS injury prevention equipment”
S17. Brac*
S18. S7 OR S8 OR S9 OR S10 OR S11 OR S12 OR S13 OR S14 OR S15 OR S16 OR S17
S19. DE “TEENAGERS” OR DE “YOUTH” OR DE “CHILDREN” OR DE “PEDIATRICS”
S20. Adolesc* or Teen* or Youth* or Young*
S21. Child* OR P#ediatric*
S22. S19 OR S20 OR S21
S23. DE “WOMEN” OR DE “WOMEN & sports” OR DE “ATHLETES” OR DE “WOMEN athletes”
S24. Female* OR Athlete* OR Female Athlete*
S25. S23 OR S24
S26. AB ((meta or synthesis or literature)) OR SU randomized OR AB published OR (meta-analysis or systematic review or review) OR AB extraction OR SU trials OR SU controlled OR AB ((search or MEDLINE or selection or sources or trials or review)) OR AB ((articles or reviewed)) OR AB ((english or language))
S27. S6 AND S18 AND S22 AND S25
S28. S26 AND S27

### Appendix A.5. CINAHL Search Terms

S1. (MH “Anterior Cruciate Ligament Injuries”)
S2. (Anterior Cruciate Ligament N1 (Injur* or tear or rupture))
S3. (ACL N1 (Injur* or tear or rupture))
S4. (MH “Knee Injuries”)
S5. Knee Injur*
S6. S1 OR S2 OR S3 OR S4 OR S5
S7. Injury prevent*
S8. Prevent* OR Intervention*
S9. Program* or Programme*
S10. (MH “Therapeutic Exercise”) OR ((MH “Physical Education and Training”)) OR (MH “Athletic Training Programs”)
S11. (MH “Plyometrics”) OR (MH “Balance Training, Physical”) OR (MH “Resistance Training”) OR (MH “Stretching”) OR (MH “Warm-Up Exercise”)
S12. Training*
S13. Propriocept* or Plyometric* or Stretch* or Resistance* or Warm-up* or Neuromuscular* or Strength* or Condition* or Agility*
S14. (MH “Education”)
S15. Educat*
S16. (MH “Orthoses”)
S17. Brac*
S18. S7 OR S8 OR S9 OR S10 OR S11 OR S12 OR S13 OR S14 OR S15 OR S16 OR S17
S19. (MH “Adolescence”) OR (MH “Child”)
S20. Adolesc* OR Teen* OR Youth* OR Young* OR Child* OR P?ediatric*
S21. S19 OR S20
S22. (MH “Female”)
S23. (MH “Athletes”)
S24. Female* OR Athlete* OR Female Athlete*
S25. S22 OR S23 OR S24
S26. AB (meta or synthesis or literature) OR MW randomized OR AB published OR PT (meta-analysis or systematic review) OR AB extraction OR MW (trials or controlled) OR AB (search or MEDLINE or selection or sources or trials or review) OR PT review OR AB (articles or reviewed) OR AB (english or language)
S27. S6 AND S18 AND S21 AND S25
S28. S26 AND S27

### Appendix A.6. PEDro Search Terms

“anterior cruciate ligament” injur* prevent*

Method Limit = systematic reviews

### Appendix A.7. Web of Science Core Collection Search Terms

1. TS = (Anterior Cruciate Ligament NEAR (Injur* or tear or rupture))
2. TS = (ACL NEAR (Injur* or tear or rupture))
3. TS = Knee Injur*
4. #3 OR #2 OR #1
5. TS = (Prevent* or Intervention*)
6. TS = (Program* or Programme*)
7. TS = Training*
8. TS = (Propriocept* or Plyometric* or Stretch* or Resistance* or Warm-up* or Neuromuscular* or Strength* or Condition* or Agility*)
9. TS = Educat*
10. TS = Brac*
11. #10 OR #9 OR #8 OR #7 OR #6 OR #5
12. TS = (Adolesc* or Teen* or Youth* or Young*)
13. TS = Child*
14. TS = P$ediatric*
15. #14 OR #13 OR #12
16. TS = Female*
17. TS = (Athlete* or Female Athlete*)
18. #17 OR #16
19. #18 AND #15 AND #11 AND #4
20. AB = (meta OR synthesis OR literature)
21. KP = randomized
22. AB = published
23. TS = (meta-analysis OR systematic review)
24. AB = extraction
25. KP = (trials OR controlled)
26. AB = (search OR MEDLINE OR selection OR sources OR trials OR review)
27. TS = review
28. AB = (articles OR reviewed) OR AB = (english OR language)
29. #28 OR #27 OR #26 OR #25 OR #24 OR #23 OR #22 OR #21 OR #20
30. #29 AND #19

### Appendix A.8. Epistemonikos Search Terms

(title:((title:(Anterior Cruciate Ligament AND (Injur* OR tear OR rupture)) OR abstract:(Anterior Cruciate Ligament AND (Injur* OR tear OR rupture))) OR (title:(ACL AND (Injur* OR tear OR rupture)) OR abstract:(ACL AND (Injur* OR tear OR rupture))) OR (title:(Knee Injur*) OR abstract:(Knee Injur*))) OR abstract:((title:(Anterior Cruciate Ligament AND (Injur* OR tear OR rupture)) OR abstract:(Anterior Cruciate Ligament AND (Injur* OR tear OR rupture))) OR (title:(ACL AND (Injur* OR tear OR rupture)) OR abstract:(ACL AND (Injur* OR tear OR rupture))) OR (title:(Knee Injur*) OR abstract:(Knee Injur*)))) AND (title:((title:(Prevent* OR Intervention*) OR abstract:(Prevent* OR Intervention*)) OR (title:(Program* OR Programme*) OR abstract:(Program* OR Programme*)) OR (title:(Training*) OR abstract:(Training*)) OR (title:(Propriocept* OR Plyometric* OR Stretch* OR Resistance* OR Warm-up* OR Neuromuscular* OR Strength* OR Condition* OR Agility*) OR abstract:(Propriocept* OR Plyometric* OR Stretch* OR Resistance* OR Warm-up* OR Neuromuscular* OR Strength* OR Condition* OR Agility*)) OR (title:(Educat*) OR abstract:(Educat*)) OR (title:(Brac*) OR abstract:(Brac*))) OR abstract:((title:(Prevent* OR Intervention*) OR abstract:(Prevent* OR Intervention*)) OR (title:(Program* OR Programme*) OR abstract:(Program* OR Programme*)) OR (title:(Training*) OR abstract:(Training*)) OR (title:(Propriocept* OR Plyometric* OR Stretch* OR Resistance* OR Warm-up* OR Neuromuscular* OR Strength* OR Condition* OR Agility*) OR abstract:(Propriocept* OR Plyometric* OR Stretch* OR Resistance* OR Warm-up* OR Neuromuscular* OR Strength* OR Condition* OR Agility*)) OR (title:(Educat*) OR abstract:(Educat*)) OR (title:(Brac*) OR abstract:(Brac*)))) AND (title:((title:(Adolesc* OR Teen* OR Youth* OR Young*) OR abstract:(Adolesc* OR Teen* OR Youth* OR Young*)) OR (title:(Child*) OR abstract:(Child*)) OR (title:(Pediatric* OR Paediatric) OR abstract:(Pediatric* OR Paediatric))) OR abstract:((title:(Adolesc* OR Teen* OR Youth* OR Young*) OR abstract:(Adolesc* OR Teen* OR Youth* OR Young*)) OR (title:(Child*) OR abstract:(Child*)) OR (title:(Pediatric* OR Paediatric) OR abstract:(Pediatric* OR Paediatric)))) AND (title:((title:(Female*) OR abstract:(Female*)) OR (title:(Athlete* OR Female Athlete*) OR abstract:(Athlete* OR Female Athlete*))) OR abstract:((title:(Female*) OR abstract:(Female*)) OR (title:(Athlete* OR Female Athlete*) OR abstract:(Athlete* OR Female Athlete*)))).

### Appendix A.9. ProQuest Dissertations and Theses Global Search Terms

((MAINSUBJECT.EXACT(“Injury prevention”) OR noft(Prevent* OR Intervention*) OR noft((Program* OR Programme*)) OR (MAINSUBJECT.EXACT(“Fitness training programs”) OR MAINSUBJECT.EXACT(“Physical education”) OR MAINSUBJECT.EXACT(“Training”) OR MAINSUBJECT.EXACT(“Sports training”)) OR (MAINSUBJECT.EXACT(“Strength training”) OR MAINSUBJECT.EXACT(“Jumping”) OR MAINSUBJECT.EXACT(“Stretching”) OR MAINSUBJECT.EXACT(“Warm up (exercise)”)) OR noft(Training*) OR noft(Propriocept* OR Plyometric* OR Stretch* OR Resistance* OR Warm-up* OR Neuromuscular* OR Strength* OR Condition* OR Agility*) OR MAINSUBJECT.EXACT(“Education”) OR noft(Educat* OR Brac*) OR MAINSUBJECT.EXACT(“Orthopedic apparatus”)) AND (MAINSUBJECT.EXACT(“Females”) OR noft(Female*) OR MAINSUBJECT.EXACT(“Athletes”) OR noft(Athlete*) OR noft(Female Athlete*)) AND ((MAINSUBJECT.EXACT(“Teenagers”) OR MAINSUBJECT.EXACT(“Children & youth”)) OR noft(Adolesc*) OR noft(Teen*) OR noft(Youth*) OR noft(Young*) OR noft(Child*) OR MAINSUBJECT.EXACT(“Pediatrics”) OR noft(P?ediatric*)) AND (noft((Anterior Cruciate Ligament NEAR/1 (Injur* OR tear OR rupture))) OR noft((ACL NEAR/1 (Injur* OR tear OR rupture))) OR noft(Knee Injur*))) AND (ab((meta OR synthesis OR literature)) OR su(randomized) OR ab((published OR extraction)) OR ft((meta-analysis OR systematic review OR review)) OR su((trials OR controlled)) OR ab((search OR MEDLINE OR selection OR sources OR trials OR review)) OR ab((articles OR reviewed)) OR ab((english OR language))).

### Appendix A.10. TRIP Database Search Terms

((Anterior Cruciate Ligament injur* OR Anterior Cruciate Ligament rupture* OR Anterior Cruciate Ligament tear*) OR (ACL injury* OR ACL rupture* OR ACL tear*) OR (Knee Injur*))(Prevent* OR Intervention* OR Program* OR Programme* OR Training* OR Propriocept* OR Plyometric* OR Stretch* OR Resistance* OR Warm-up* OR Neuromuscular* OR Strength* OR Condition* OR Agility* OR Educat* OR Brac*)(Adolesc* OR Teen* OR Youth* OR Young* OR Child* OR Pediatric* OR Paediatric*)(Female* OR Athlete* OR Female Athlete*).

## Appendix B

**Table A1:** The reasons for the exclusion of all studies that were excluded after a full-text review are listed below.

Study	Main Reason for Exclusion
Chiaro [56]	Adult population
Albaugh et al. [57] No author [58] Halvorson [59] May et al. [60] Della Villa et al. [61]	Could not find full-text
Herman et al. [62] Dompier [63] Hespanhol Junior et al. [64] No author [65] Meyer et al. [66] Elliot et al. [67] Myer et al. [18] Hewett et al. [68] Silvers et al. [69] Sugimoto et al. [54] Sugimoto et al. [17]	Duplicate
Rishiraj et al. [70] Elliot et al. [67] Gokeler et al. [71] Hewett et al. [72] Myer et al. [73] Laible et al. [74] Myer et al. [75] Dai et al. [76] Zakieh et al. [77] Paulson et al. [78] LaBella et al. [79] Arabia et al. [80] Myer et al. [81] Padua et al. [53] Bonnette et al. [82] Nessler et al. [83] Kiefer et al. [84] Hewett et al. [68] Myer et al. [85] Silvers et al. [69] Dharamsi et al. [86] Labella et al. [87] Myer et al. [88] Bencke et al. [89] Myer et al. [90] Mehl et al. [91] Ladenhauf et al. [92] Graziano et al. [93] Kelly [94] Acevedo et al. [95] Brophy et al. [96] Bisciotti et al. [97]	Narrative review without a search algorithm
Taylor et al. [98] Chang et al. [99] Sugimoto et al. [54] Assaly et al. [100] Myer et al. [18] Sugimoto et al. [101] Ramirez et al. [102] Crossley et al. [103] Sugimoto et al. [55] Petushek et al. [104] Grimm et al. [105] Huang et al. [106]	No differentiation between mechanisms of ACL injury in results and/or analyses
Zheng et al. [107] Romero-Moraleda et al. [108]	Not in English
Herzberg et al. [109]	Wrong intervention
Willadsen et al. [110] Weber et al. [111] Trojian et al. [112] Emery et al. [113] Lim et al. [114] Ter Stege et al. [115] Barber-Westin et al. [116] Monajati et al. [117]	Wrong outcomes
Paszkewicz et al. [118] Prather [119] Barber-Westin et al. [120] No author [121] Bottino [122] Moksnes et al. [123] Hornbeck et al. [124] Halvorson [125] Powers et al. [126] Howe [127] Brunner et al. [24] Garcia [128] Nagano et al. [129] Queen [130]	Wrong study design

**Table A2:** The reasons for the exclusion of all studies that were excluded after a full-text review of primary literature are listed below.

Study	Main Reason for Exclusion
Lewis et al. [131] Jari et al. [132] Sibbala et al. [133] Owoeye et al. [134] Richmond et al. [135]	Wrong outcomes
Paulson et al. [79] Halvorson [126] Potera [136] No author [122] Murray et al. [137] Decker et al. [138]	Wrong study design
Foss et al. [139]	No differentiation between mechanisms of ACL injury
Omi et al. [140]	Adult population
Achenbach et al. [31]	Duplicate
